# Supplementary material for: DNA Polymerase Theta Plays a Critical Role in Pancreatic Cancer Development and Metastasis
Source: Cancers (Basel). 2022 Aug 23;14(17):4077. doi: 10.3390/cancers14174077 (PMC9454495; doi:10.3390/cancers14174077)
Supplement: Supplementary file 1 [file cancers-14-04077-s001.zip › Supplementary Figures_after revision_NEW.pdf]

## Supplementary Figure S1

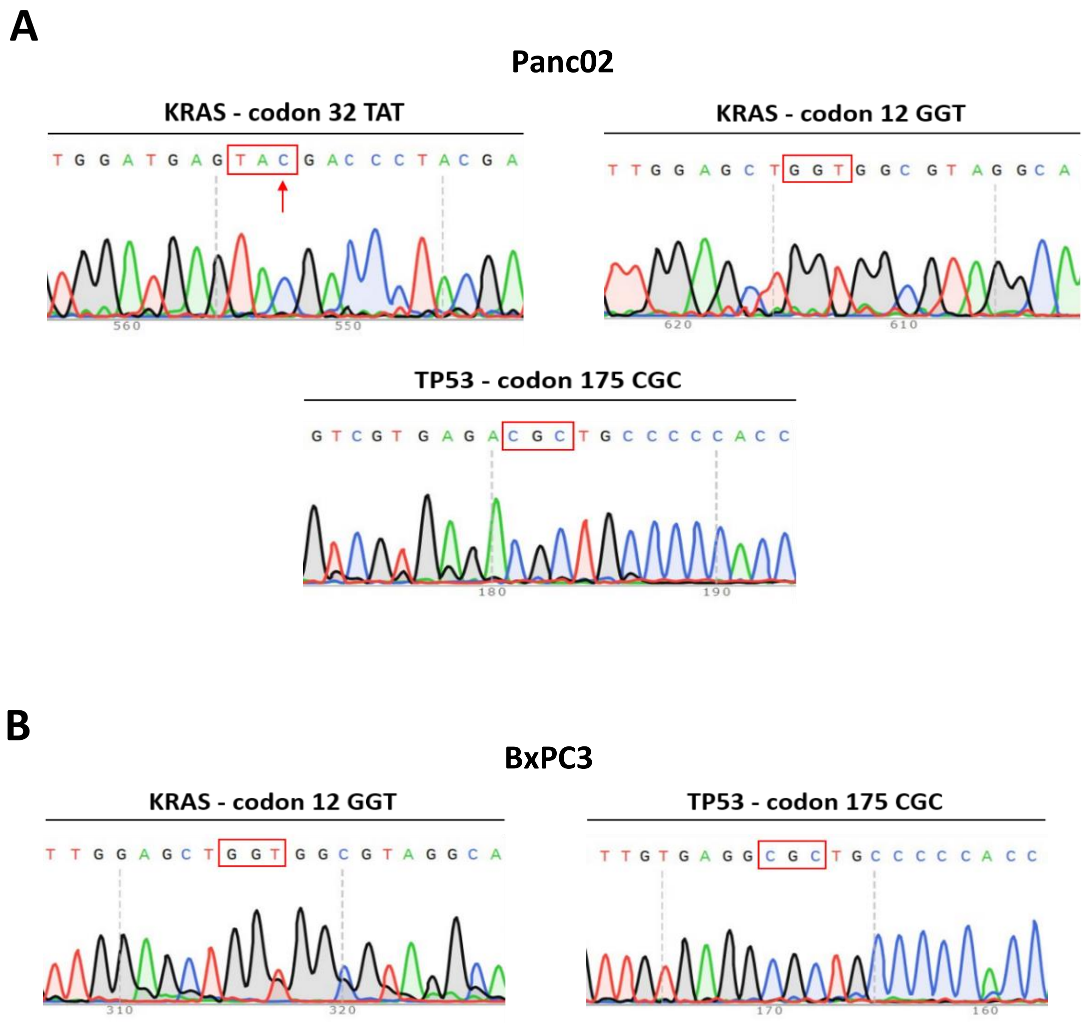


**Supplementary Figure S1. Detection of mutations in *KRAS* exon 2, *TP53* exon 5 in pancreatic cancer cell lines.** **(A)** Representative sequence analysis for each case in Panc02 mouse pancreatic cancer cell line is presented. The rectangle contains the codons in which hotspot mutations were expected. The arrow indicates the synonymous SNP in codon 32 of *KRAS* gene. **(B)** Representative sequence analysis for each case in BxPC3 human pancreatic cancer cell line is presented. No somatic mutations in KRAS and TP53 genes were found in this pancreatic cancer cell line.

## Supplementary Figure S2

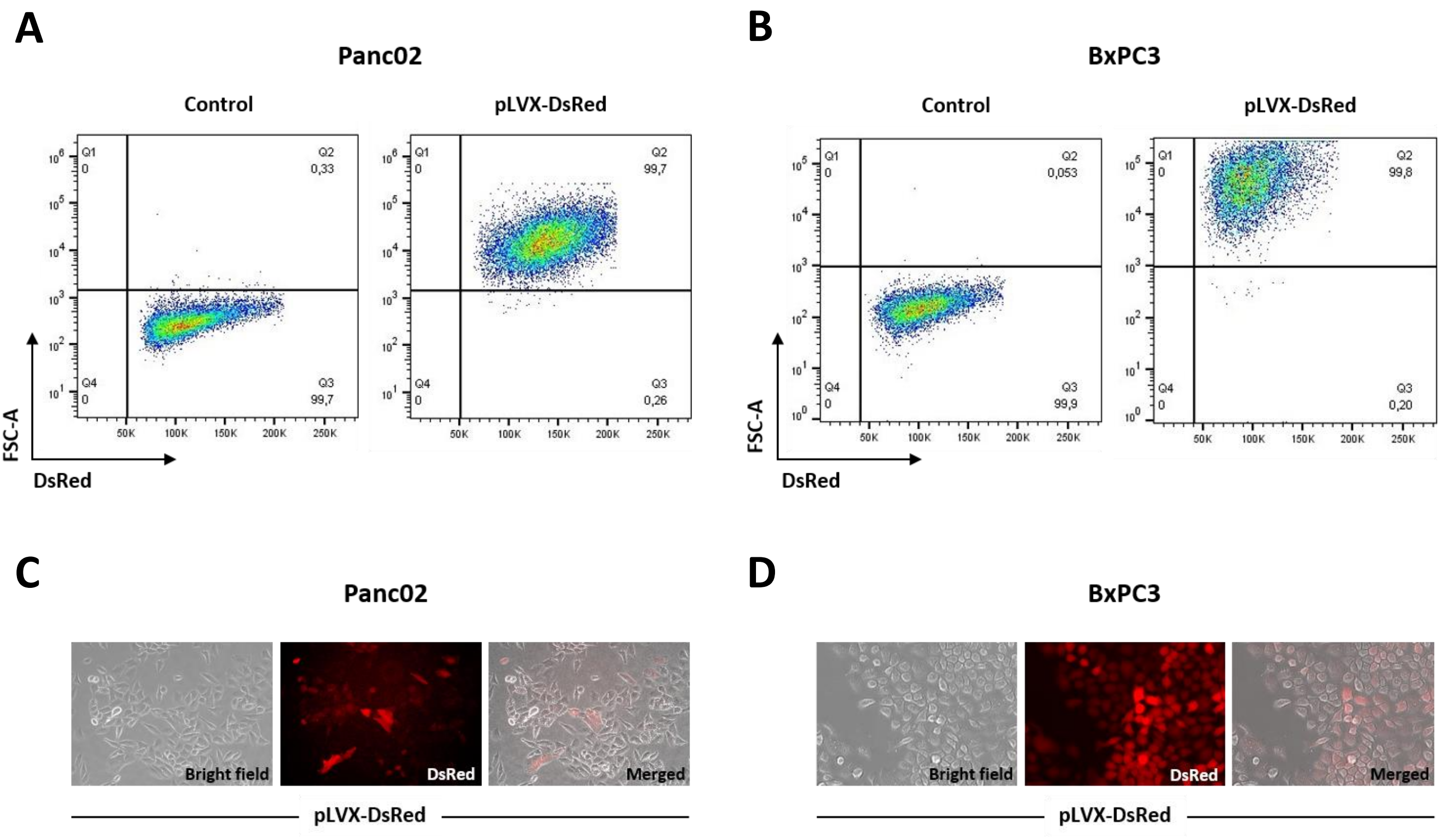


**Supplementary Figure S2. Lentiviral transduction of pLVX-DsRed, KRAS wild-type and mutagenic KRAS plasmids in pancreatic cancer cell lines. (A-B)** The transduction efficiency of pLVX-DsRed plasmid in **(A)** Panc02 and **(B)** BxPC3 cell line was demonstrated by flow cytometry and microscopic analysis. **(C-D)** Representative images of **(C)** Panc02 and **(D)** BxPC3 cells expressing pLVX-DsRed plasmid.

## Supplementary Figure S3

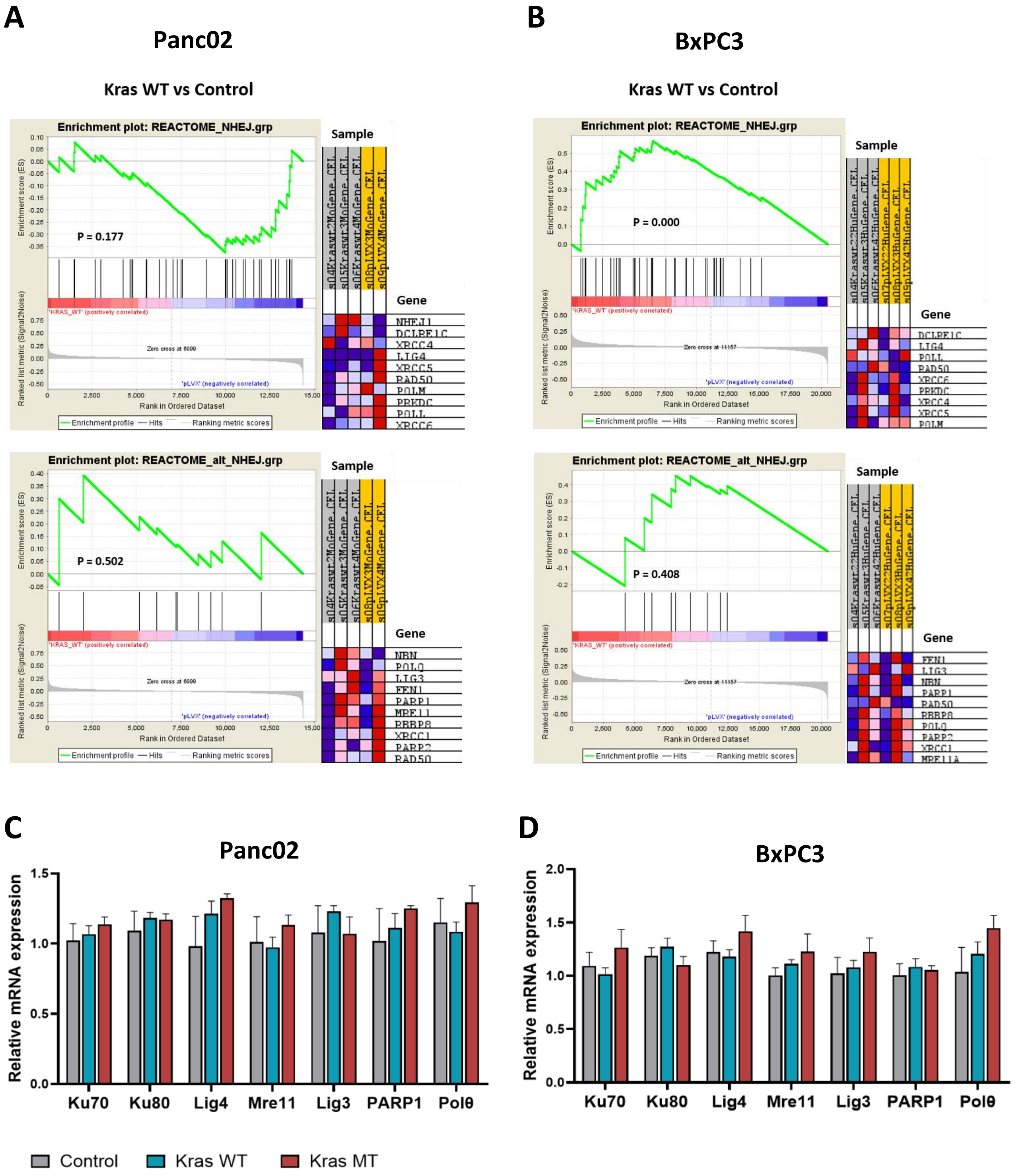


**Supplementary Figure S3. Kras does not regulate the expression level of alt-EJ components at the transcriptional level. (A-B)** Gene Set Enrichment Analysis plots for the Reactome NHEJ and alt-EJ in Panc02 and BxPC3 cells are shown. The heatmap on the right side of each panel visualizes the genes contributing the most to the enriched pathway. The green curve corresponds to the ES (enrichment score) curve, the running sum of the weighted enrichment score in GSEA. P value are reported within each graph (Panc02 control, n=2; Panc02 KrasWT, n=3; BxPC3 control, n=3; KrasWT, n=3). **(C-D)** mRNA expression of alt-EJ and c-NHEJ components in Panc02 and BxPC3 cells upon exogenous expression of KrasWT and mutagenic $Kras^{G12D}$. Relative mRNA levels were normalized to 5S RNA of alt-EJ and c-NHEJ factors from three independent experiments (mean ± SD; **P* < 0.05 is considered as significant; Student's t-test).

## Supplementary Figure S4

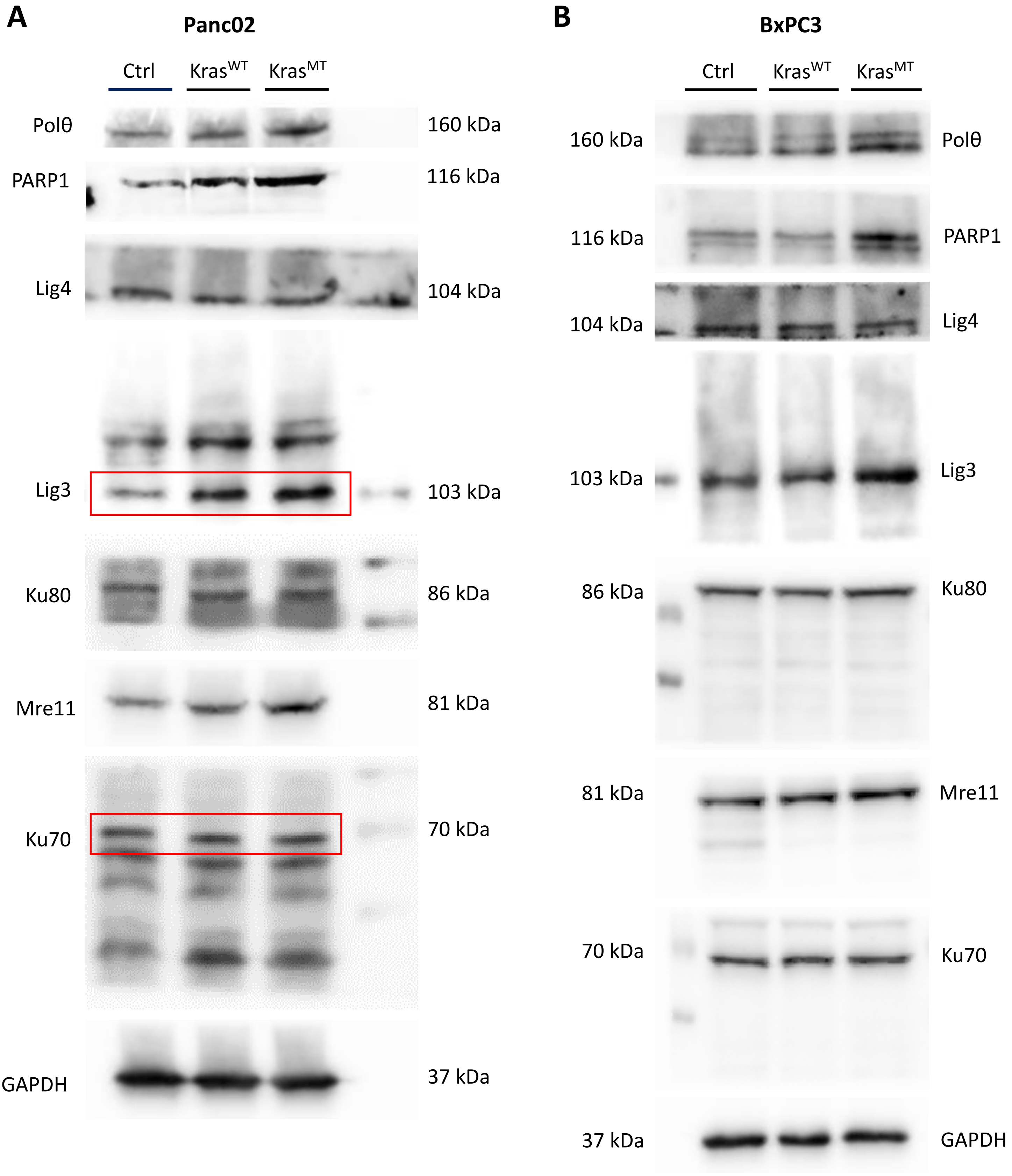


**C**

| **Panc02** | Control | Kras WT | Kras MT |
|---|---|---|---|
| Polθ/GAPDH | 1 | 2,129 | 4,109 |
| PARP1/GAPDH | 1 | 3,361 | 4,999 |
| Lig4/GAPDH | 1 | 0,865 | 0,931 |
| Lig3/GAPDH | 1 | 3,745 | 4,625 |
| Ku80/GAPDH | 1 | 0,947 | 0,976 |
| Mre11/GAPDH | 1 | 1,613 | 2,375 |
| Ku70/GAPDH | 1 | 0,987 | 1,093 |

| **BxPC3** | Control | Kras WT | Kras MT |
|---|---|---|---|
| Polθ/GAPDH | 1 | 1,603 | 2,662 |
| PARP1/GAPDH | 1 | 1,341 | 2,862 |
| Lig4/GAPDH | 1 | 1,149 | 1,067 |
| Lig3/GAPDH | 1 | 1,271 | 1,946 |
| Ku80/GAPDH | 1 | 1,038 | 1,282 |
| Mre11/GAPDH | 1 | 1,342 | 1,512 |
| Ku70/GAPDH | 1 | 1,101 | 1,174 |

**Supplementary Figure S4. Whole western blot and densitometry readings/intensity ratio of alt-EJ and c-NHEJ components. (A-B)** Western blot analysis of total lysate from transduced **(A)** Panc02 and **(B)** BxPC3 cells. The level of Polθ, PARP1, Lig4, Lig3, Ku80, Mre11 and Ku70 were analyzed. GAPDH was used as a loading control. Red rectangles are the selected for figure 1. **(C)** Densitometry readings/intensity ratio of each band of total blot from Panc02 and BxPC3 cells.

## Supplementary Table S1

| ID | Strain | Gender | Age (days) | PDA | Metastasis | Other |
|---|---|---|---|---|---|---|
| **873** | KC | F | 289 | N | N | |
| **931** | KC | F | 164 | N | N | |
| **938** | KC | M | 96 | N | N | |
| **1034** | KC | M | 161 | N | N | |
| **1161** | KC | M | 132 | Y | N | |
| **872** | KC | M | 440 | N | N | |
| **878** | KC | M | 431 | N | N | |
| **916** | KC | M | 400 | N | N | |
| **1003** | KC | M | 326 | Y | N | |
| **251** | KC | M | 258 | N | N | |
| **208** | KC | F | 286 | Y | Y' liver | Pancreatobiliary type IPMN |
| **1151** | KC | F | 209 | Y | N | Sarcomatoid dedifferentiation, Fatty Infiltration (FI) |
| **957** | KC | M | 412 | N | N | |
| **967** | KC | M | 407 | N | N | |
| **994** | KC | M | 413 | N | N | |
| **1033** | KC | F | 440 | Y | N | Sarcomatoid |
| **1099** | KC | M | 440 | Y | N | Serous microcystic adenoma |
| **1116** | KC | M | 420 | N | N | |
| **1117** | KC | M | 420 | N | N | |
| **1126** | KC | F | 418 | N | N | Serous microcystic adenoma |
| **1050** | qKC | F | 274 | Y | Y' liver, lung | Sarcomatoid, Fatty Infiltration (FI) |
| **990** | qKC | M | 397 | Y | N | |
| **959** | qKC | F | 440 | N | N | Serous microcystic adenoma |
| **960** | qKC | F | 440 | N | N | |
| **1039** | qKC | F | 387 | Y | Y' liver | Sarcomatoid, Fatty Infllltration (FI) |
| **981** | qKC | F | 440 | Y | N | |
| **988** | qKC | M | 440 | N | N | |
| **1041** | qKC | M | 404 | Y | N | |
| **1080** | qKC | F | 360 | Y | N | |
| **1013** | qKC | F | 440 | N | N | |
| **1090** | qKC | M | 375 | Y | Y' liver | |
| **1046** | qKC | F | 446 | N | N | |
| **1086** | qKC | F | 440 | Y | N | |
| **1089** | qKC | M | 424 | Y | Y' liver, Y' lung | Sarcomatoid, Fatty Infiltration (FI) |

**Supplementary Table S1. Clinical spectrum of disease in KC and qKC mice.** Histopathological analysis of animals used in the survival curve (N = no, Y = yes).
